# Programmed Evolution for Optimization of Orthogonal Metabolic Output in Bacteria

**DOI:** 10.1371/journal.pone.0118322

**Published:** 2015-02-25

**Authors:** Todd T. Eckdahl, A. Malcolm Campbell, Laurie J. Heyer, Jeffrey L. Poet, David N. Blauch, Nicole L. Snyder, Dustin T. Atchley, Erich J. Baker, Micah Brown, Elizabeth C. Brunner, Sean A. Callen, Jesse S. Campbell, Caleb J. Carr, David R. Carr, Spencer A. Chadinha, Grace I. Chester, Josh Chester, Ben R. Clarkson, Kelly E. Cochran, Shannon E. Doherty, Catherine Doyle, Sarah Dwyer, Linnea M. Edlin, Rebecca A. Evans, Taylor Fluharty, Janna Frederick, Jonah Galeota-Sprung, Betsy L. Gammon, Brandon Grieshaber, Jessica Gronniger, Katelyn Gutteridge, Joel Henningsen, Bradley Isom, Hannah L. Itell, Erica C. Keffeler, Andrew J. Lantz, Jonathan N. Lim, Erin P. McGuire, Alexander K. Moore, Jerrad Morton, Meredith Nakano, Sara A. Pearson, Virginia Perkins, Phoebe Parrish, Claire E. Pierson, Sachith Polpityaarachchige, Michael J. Quaney, Abagael Slattery, Kathryn E. Smith, Jackson Spell, Morgan Spencer, Telavive Taye, Kamay Trueblood, Caroline J. Vrana, E. Tucker Whitesides

**Affiliations:** 1 Department of Biology, Missouri Western State University, Saint Joseph, Missouri, United States of America; 2 Department of Biology, Davidson College, Davidson, North Carolina, United States of America; 3 Department of Mathematics and Computer Science, Davidson College, Davidson, North Carolina, United States of America; 4 Department of Computer Science, Math and Physics, Missouri Western State University, Saint Joseph, Missouri, United States of America; 5 Department of Chemistry, Davidson College, Davidson, North Carolina, United States of America; Tsinghua University, CHINA

## Abstract

Current use of microbes for metabolic engineering suffers from loss of metabolic output due to natural selection. Rather than combat the evolution of bacterial populations, we chose to embrace what makes biological engineering unique among engineering fields – evolving materials. We harnessed bacteria to compute solutions to the biological problem of metabolic pathway optimization. Our approach is called *Programmed Evolution* to capture two concepts. First, a population of cells is *programmed* with DNA code to enable it to compute solutions to a chosen optimization problem. As analog computers, bacteria process known and unknown inputs and direct the output of their biochemical hardware. Second, the system employs the *evolution* of bacteria toward an optimal metabolic solution by imposing fitness defined by metabolic output. The current study is a proof-of-concept for Programmed Evolution applied to the optimization of a metabolic pathway for the conversion of caffeine to theophylline in *E. coli*. Introduced genotype variations included strength of the promoter and ribosome binding site, plasmid copy number, and chaperone proteins. We constructed 24 strains using all combinations of the genetic variables. We used a theophylline riboswitch and a tetracycline resistance gene to link theophylline production to fitness. After subjecting the mixed population to selection, we measured a change in the distribution of genotypes in the population and an increased conversion of caffeine to theophylline among the most fit strains, demonstrating Programmed Evolution. Programmed Evolution inverts the standard paradigm in metabolic engineering by harnessing evolution instead of fighting it. Our modular system enables researchers to program bacteria and use evolution to determine the combination of genetic control elements that optimizes catabolic or anabolic output and to maintain it in a population of cells. Programmed Evolution could be used for applications in energy, pharmaceuticals, chemical commodities, biomining, and bioremediation.

## Introduction

### Metabolic Engineering

For many years, naturally occurring metabolism has been harnessed by metabolic engineering. Anabolic pathways have been adapted for the conversion of readily available, inexpensive starting materials into proteins or metabolites with important uses such as biofuels, construction materials, pharmaceuticals, and commodity chemicals [1]. Catabolic pathways can be used for bioremediation and biomining [2]. Cloning of genes or entire metabolic pathways from rare or genetically intractable organisms into well-known and safe microorganisms such as *E*. *coli* has advanced the field of metabolic engineering, but sustaining metabolic output has been challenging. A variety of related approaches to metabolic engineering have produced initial success but maintenance of metabolic output has been challenging. Traditionally, metabolic engineers have attempted to maximize metabolic output by what has been called a “rational approach” [3]. A quantitative understanding of naturally occurring or synthetic gene expression control elements encouraged the development of software for optimization of metabolic pathways [4–6]. This type of forward engineering is limited by the choice of parts used in synthetic circuits and our incomplete understanding of bacterial physiology. A “rationally irrational” approach takes advantage of selection and evolution [3]. For example, bottlenecks in the biosynthesis of a sesquiterpene were identified by mass spectrometry and fine-tuned by codon optimization and use of a stronger promoter [7]. Another approach to maximizing metabolic output used riboswitch metabolite biosensors to conduct high throughput screens of libraries of alleles coding for pathway enzymes [8]. The investigators used directed evolution to produce a caffeine demethylase as a proof-of-concept for optimization of coding sequence. Global Transcription Machinery Engineering (gTME) mutates transcription factor genes in an effort to adjust the transcription of heterologous genes compared to host genes [9]. Selection of the desired outcome was achieved by increased ethanol tolerance. Tunable Intergenic Regions (TIGR) boosted mevalonate production via synthetic RNA constructs that coupled the transcription of two genes and enabled screening for improved translation ratios [10]. Multiplex Automated Genome Engineering (MAGE) increased lycopene production in *E*. *coli* by introducing alternative RBS elements into four pathway genes with the simultaneous introduction of nonsense mutations into genes in competing pathways [11]. Engineered strains were screened for enhanced accumulation of the red lycopene pigment. In their 2012 review of metabolic engineering, Boyle and Silver (2012) noted that a common problem in all these approaches is the use of an *ad hoc* screening or selection strategy that is particular to the pathway involved [3]. They noted, “The lack of generalized methods for pathway screening and selection limits the broad application of combinatorial pathway optimization methods” [3]. Synthetic biology lacks a generalized method for pathway selection that could find widespread applications in metabolic engineering.

Several research groups have tested new methods to increase orthogonal metabolic output in bacteria. Li *et al*. used Multiplex Iterative Plasmid Engineering (MIPE) to introduce mutations in genes that control biosynthesis [12]. Multichange Isothermal mutagenesis was developed by Mitchell *et al*. and could be used to introduce variation in genetic circuits controlling orthogonal metabolism in bacteria [13]. Du *et al*. invented Customized Optimization of Metabolic Pathways by Combinatorial Transcriptional Engineering (COMPACTER), which introduces variation in the strength of promoters controlling metabolic pathway genes and enables high-throughput screening and selection for metabolic pathway enhancement [14]. Zelcbuch *et al*. reported a method for combinatorially pairing genes with ribosome binding sites in order to stimulate the desired bacterial metabolism [15]. A system of using transcription factors that control expression of antibiotic resistance genes was used by Dietrich *et al*. to select variants with amplified metabolic pathway output [16]. Finally, Yang *et al*. (2013) developed a system that used an RNA riboswitch connected to a gene encoding tetracycline resistance to increase the native metabolic output of lysine in *E*. *coli* [17]. Programmed Evolution differs from all these efforts as described below.

### Programmed Evolution for Optimization of Metabolic Pathways

**Fig 1:**
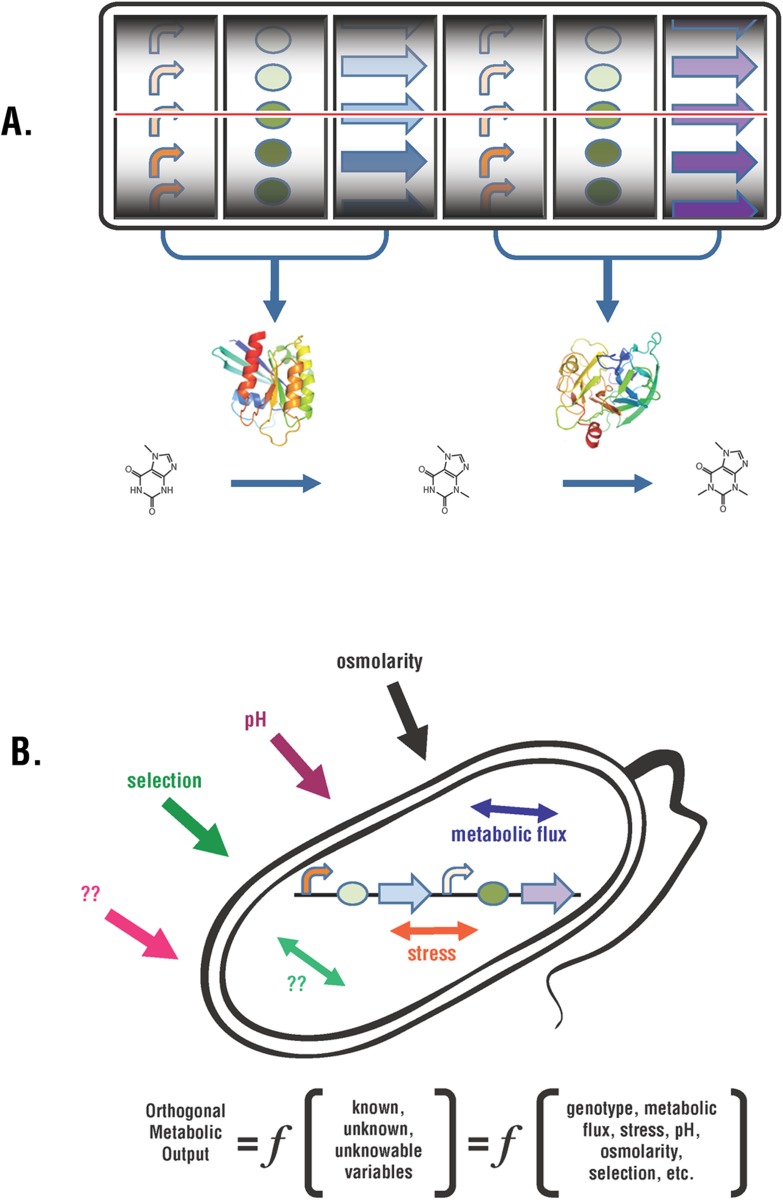
Optimization of Metabolic Pathways. (A) Orthogonal metabolic output in a bacterial cell is depicted as a function (*f*) of the genetic circuit controlling metabolism and additional variables. (B) Two gene expression cassettes are drawn that encode enzymes controlling a metabolic pathway. Promoters, ribosome binding sites, and alleles for the two cassettes are chosen from a library of elements.

We view the challenge of optimizing orthogonal metabolic pathways in a bacterial cell as a computational problem. Bacterial cells are computational machines capable of using a variety of chemical and physical inputs and processing them with gene expression algorithms to regulate their biochemical hardware. As illustrated in Fig. 1A, we suggest that the orthogonal metabolic output is a function of some variables that we know, some that are unknown, and some that are unknowable to us. Variables that affect cell growth include pH and osmolarity of the media, energy requirements for growth and division, stress caused by orthogonal metabolism, and endogenous metabolic flux. The variable that we want to manipulate is the genotype controlling the orthogonal metabolic pathway. Fig. 1B shows two gene expression cassettes encoding two enzymes that control a two-step metabolic pathway. Each pathway can be altered with families of promoters and RBS elements of varying strengths, and families of alleles for each of the genes depicted. Even when the families of control elements and alleles are small, the number of possible genotypes is large since it is the product of the number of members in each family. We cannot know *a priori* which combination of all the significant known and unknown genetic variables would produce the optimal sustained output from a given metabolic pathway.

We leveraged our previous experience in programming bacterial computers that solve mathematical problems [18–22] to program bacteria to solve the biological problem of optimal sustained orthogonal metabolism. We call our approach *Programmed Evolution* in order to capture two important concepts. First, we *program* a population of cells with DNA code to compute solutions to an optimization problem of our choosing. Bacteria continuously gather inputs (known and unknown to us) for their calculations, including the DNA sequence context of genetic control elements, the molecular interaction of parts and devices, the energy requirements for heterologous and endogenous metabolism, the effects of metabolic flux, and the changing genomic context of the population of cells [23]. Bacteria process all of this information as living analog computers, using the results to direct the operation of their biochemical hardware. The bacteria are better informed and more capable of making these calculations than people and the silicon computers they program with incomplete information and models. The second important concept we employ is *evolution* of a bacterial population toward solutions to the problem of optimizing a metabolic pathway. Programmed Evolution introduces genetic variation to a bacterial population and imposes selection on it. We tie biological fitness to the ability to balance optimal production of the desired product with cell growth and division. After one cycle of Programmed Evolution, the allele frequency in the population will have changed, which is the definition of evolution. In successive cycles, programmed genetic variation in the form of diverse genetic elements is subjected to further selection and the population continues to evolve. Over time, the population evolves an optimal metabolic solution since survival depends upon increased production of the desired product and maintenance of cellular physiology. Coupling fitness to optimal metabolic output prevents the population from evolving away from optimal product formation.

**Fig 2:**
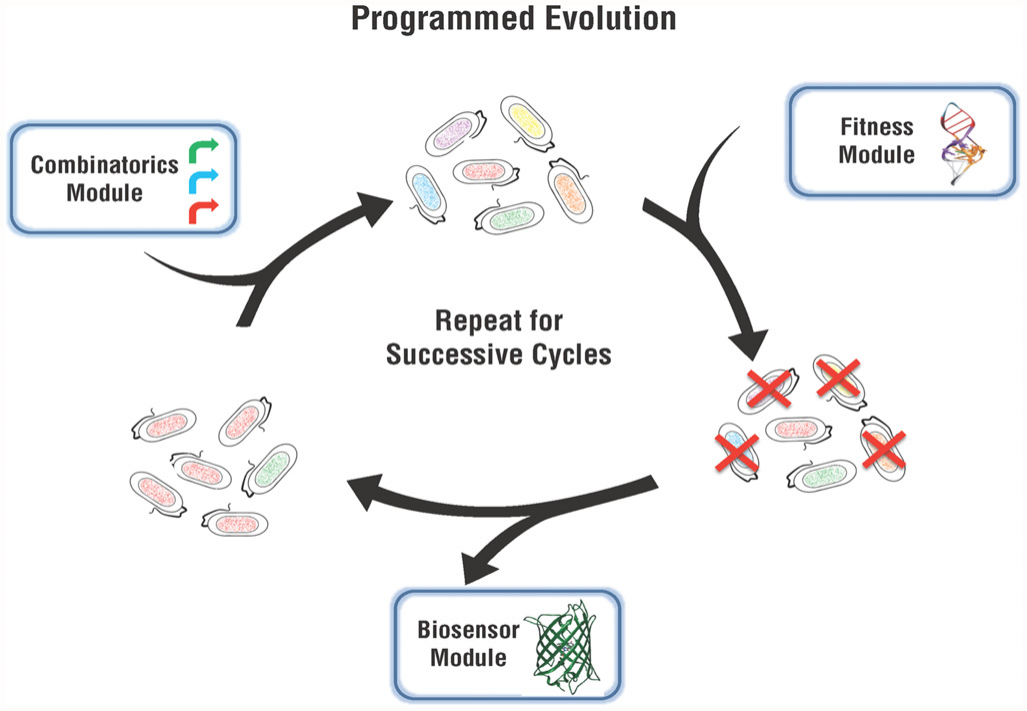
Programmed Evolution. The Combinatorics Module facilitates variation of elements controlling orthogonal metabolism. Genetic variation is illustrated by different colors of bacteria. The Fitness Module defines fitness as orthogonal metabolic output and cell growth, and imposes negative selection on bacteria with low metabolic output, shown by elimination of some of the colored bacteria. A Biosensor Module is used to measure the metabolic output of the population or individual cells. Programmed Evolution can be repeated for successive cycles.

We modularized Programmed Evolution of orthogonal metabolism by a bacterial population as three separate functions: 1) generating genetic diversity in a bacterial population; 2) linking fitness to optimal metabolic output; and 3) measuring metabolic output. Programmed Evolution uses a Combinatorics Module to efficiently generate genetic diversity in the population, a Fitness Module to link metabolic product formation to cell survival, and a Biosensor Module to measure the desired output (Fig. 2). The Combinatorics Module makes it easy to simultaneously introduce parts from libraries of promoters, ribosome binding sites, alleles, degradation tags, transcriptional terminators, and origins of replication, all of which will influence the output of the orthogonal metabolic pathway. Libraries of parts are introduced using our adaptation of Golden Gate Assembly [24] called Junction Golden Gate Assembly (J-GGA) and our online tool Golden Gate Assembly Junction Evaluative Tool (GGAJET) that facilitates J-GGA design [25]. Using the Combinatorics Module, each genetic variable could be tested in succession for step-wise optimization if the total number of combination is too large to clone. The key to Programmed Evolution is its Fitness Module, which uses a riboswitch that binds the desired metabolic product. The riboswitch controls production of a protein that increases the fitness of bacteria that produce the desired metabolite. The riboswitch used in the Fitness Module can also be used in a Biosensor Module to quantify the metabolic output. When connected to a fluorescent protein, orthogonal metabolic output can be quantified in mixed populations of bacteria or individual clones. Programmed Evolution allows researchers to genetically program bacteria to execute search algorithms in high-dimensional space and compute solutions to metabolic optimization problems using natural selection. Programmed Evolution differs from the approaches developed by Du *et al*. [14], Zelcbuch *et al*. [15], Dietrich *et al*. [16] and Yang *et al*. [17] in several ways. The Combinatorics Module of Programmed Evolution allows for introduction of extensive variation in any of several elements that directly control metabolic enzymes. Programmed Evolution is focused on orthogonal metabolism, as demonstrated in the proof-of-concept reported here, instead of native metabolism. Finally, Programmed Evolution separately modularizes the processes of selection and biosensing so that new *ad hoc* methods are not required every time a different metabolite needs to be optimized.

## Materials and Methods

### Junction Golden Gate Assembly

Construction of the scaffold for Junction Golden Gate Assembly (J-GGA) began with DNA synthesis (all oligos purchased from Integrated DNA Technologies) of two top strands with the sequence 5’ AATTCGCGGCCGCTTCTAGAGCCGTATACACAGAGGACCACGTTGGG 3’ and 5’ TCTTGAACCAGGAGCGGAGTGCTAATGACTGTGCGATTAGGCCTAACGAACAGACAGCTACTAGTAGCGGCCGCTGCA 3’ and two bottom strands of the sequence 5’ GCGGCCGCTACTAGTAGCTGTCTGTTCGTTAGGCCTAATCGCAC 3’ and 5’ AGTCATTAGCACTCCGCTCCTGGTTCAAGACCCAACGTGGTCCTCTGTGTATACGGCTCTAGAAGCGGCCGCG 3’. The four oligonucleotides were mixed at 5 µM each in 20 µL of 0.1 M NaCl, 10 mM Tris-HCl pH 7.4, boiled for 4 minutes in a beaker with 400 mL H_2_0, and allowed to cool for 2 hours. The annealed oligonucleotides were ligated into a pSB1A2 vector that had the BsaI site removed (pSB1A2-BR, J119061). pSB1A2-BR was digested with EcoRI and PstI and gel purified. T4 DNA Ligase and 2X Rapid Ligation Buffer (Promega) were used for ligation. The products were transformed into JM109 *E*. *coli* (Zymo Research) and plated on LB with 50 µg/mL ampicillin. The scaffold for J-GGA is part number J119314 in the Registry of Standard Biological Parts. The J-GGA scaffold employs BsaI Golden Gate Assembly to build a gene expression cassette. The vector is amplified by inverse PCR using primers with BsaI sites that produce 5' overhanging sticky ends for the junctions involved. Assembled oligonucleotides carry complementary sticky ends. PCR products carry BsaI sites that are digested to generate complementary sticky ends. J-GGA is performed with BsaI (New England Biolabs) and T4 DNA Ligase (Promega) mixed in the same tube along with 1X T4 DNA Ligase buffer. PCR primers needed for J-GGA with the scaffold are:

*JunctionA_forward; 5’ GCATGGTCTCTCCGTATACACAGAGGACC 3’*

*JunctionA_reverse; 5’ GCATGGTCTCTGGTCCTCTGTGTATACGG 3’*

*JunctionB_forward; 5’ GCATGGTCTCTTGGGTCTTGAACCAGGAG 3’*

*JunctionB_reverse; 5’ GCATGGTCTCTCTCCTGGTTCAAGACCCA 3’*

*JunctionC_forward; 5’ GCATGGTCTCTGTGCTAATGACTGTGCGA 3’*

*JunctionC_reverse; 5’ GCATGGTCTCTTCGCACAGTCATTAGCAC 3’*

*JunctionD_forward; 5’ GCATGGTCTCTGCCTAACGAACAGACAGC 3’*

*JunctionD_reverse; 5’ GCATGGTCTCTGCTGTCTGTTCGTTAGGC 3’*

Amplification of the scaffold used 20 µL reactions including 0.1 ng of plasmid template, 1 µL of a 10 µM stock of the appropriate primer pair, and 10 µL of 2X GoTaq Green (Promega). The thermal profile was: 10 minutes at 94°C; 30 cycles of 15 seconds at 94°C, 15 seconds at 45°C, 3 minutes at 74°C; 5 minutes at 74°C. Preparation of the P5 promoter (J119031) for J-GGA involved annealing of the oligonucleotides P5_top 5’ GACCTTGACAATTAATCATCCGGCTCGTAATTTATGTGGA 3’ and P5_Bottom 5’ CCCATCCACATAAATTACGAGCCGGATGATTAATTGTCAA 3’ as described above. The BD18 C dog RBS (J119024) [23] was prepared for J-GGA by annealing the oligonucleotides BD18_top 5’ GGAGGGGCCCAAGTTCACTTAAAAAGGAGATCAACAATGAAAGCAATTTTCGTACTGAAACATCTTAATCATGCGACGGAGCG 3’ and BD18_bot 5’ GCACCGCTCCGTCGCATGATTAAGATGTTTCAGTACGAAAATTGCTTTCATTGTTGATCTCCTTTTTAAGTGAACTTGGGCCC 3’. For use in J-GGA, the RFP gene was amplified from part J04450 using PCR primers JGGA2.0_RFP_for 5’ GCATGGTCTCTGCGAGCTTCCTCCGAAGACGTTATC 3’ and JGGA2.0_RFP_rev 5’ GCATGGTCTCAAGGCTTATTAAGCACCGGTGGAG 3’. J-GGA was carried out in 10 µL reactions containing 50 ng amplified plasmid, an equimolar quantity of the desired insert, 1 µL 10 T4 DNA Ligase buffer (Promega), 0.5 µL (10 units) BsaI (New England Biolabs), and 0.5 µL (1 Weiss unit) T4 DNA Ligase (Promega). Reactions were placed in a thermal cycler for 20 cycles of 1 minute at 37°C; 1 minutes at 16°C followed by 15 minutes at 37°C. Products were transformed into JM109 *E*. *coli* (Zymo Research) and plated on LB agar, Lennox (Accumedia, Neogen Corp.) with 50 µg/mL ampicillin.

### Quantitative PCR

To measure the plasmid copy number (PCN) using qPCR, two sets of primers were developed for amplification of plasmid and chromosomal DNA. Primers binding to the promoter region of the ampicillin resistance gene in pSB1A2 were pAMPfor 5’ cccgaaaagtgccacctga 3’ and pAmprev 5’ AATTCTGCCTCGTGATACGCCTAT 3’. Primers for amplification of the promoter region of the *E*. *coli* chromosomal DNA polymerase I gene (Accession number U00096 20) were DNAPIfor 5’ GCGAGCGATCCAGAAGATCT 3’ and DNAPIrev 5’ GGGTAAAGGATGCCACAGACA 3’. Chromosomal and plasmid DNA template was prepared by standardizing overnight cultures to and absorbance at 590 nm of 0.1, boiling 500 µL of the bacterial culture for 5 minutes in a 1.5 ml tube, and immediately placing the tube in the freezer for 30 minutes. Reactions were assembled in 20 µL using 0.5 µM of each primer, 5 dilutions of the chromosomal and plasmid DNA template (10^–2^, 10^–2.5^, 10^–3^, 10^–3.5^, and 10^–4^) and SyBR Green Master Mix (Life Technologies). The Cycle Threshold for measuring PCN of mutations was set at 0.020. The method of PCN determination was adapted from Skulj *et al*. [26]. The efficiency of chromosomal and plasmid PCR reactions was calculated using E = 10^(−1/slope)^. The slope is determined from graphs of chromosomal and plasmid dilutions versus C_t_ values. Plasmid copy number was calculated as PCN = (E_c_)(Ct_c_)/(E_p_)(Ct_p_), where E_c_ is the efficiency of chromosomal DNA replication, E_p_ is the efficiency of plasmid DNA replication, Ct_c_ is the C_t_ value of the chromosomal DNA amplification, and Ct_p_ is the C_t_ value of the plasmid DNA amplification. After the PCN was determined for each serial dilution, all PCNs were averaged to produce a mean PCN and standard deviation for each origin of replication mutation.

### Construction of Plasmids

The theophylline Fitness Module was constructed using BioBrick cloning [27]. The T5 promoter was ligated to the theophylline riboswitch D to make part J100065. GFP output of the biosensor was determined with a Biotek Synergy fluorometer set for 485 nm excitation and 515 nm emission and absorption set at 590 nm. The *tetA* tetracycline gene (J31007) was added to make the Fitness Module (J119140). The theophylline Biosensor Module (J100079) was constructed from J100065 and the superfolder GFP gene (J100070). The caffeine demethylase gene was synthesized (GeneArt Life Technologies) with codon optimization for *E*. *coli* as eCDM8 (J100100). For use in the starting population for Programmed Evolution, J119346 contains a high strength promoter (J119031), high strength RBS (J119028), and the eCDM8 gene (J100100). The theophylline BioSensor Module contains a Plac (R0010) promoter and RFP (E1010). J119347 was constructed with a low strength promoter (J119030), low strength RBS (J119025), and eCDM8 (J100100). The theophylline BioSensor Module contains Plac (R0010) and GFP (E0040). The chaperone plasmids (Chaperone Plasmid Set, Clonetech Laboratories, Inc.) express three different chaperones individually and in two combinations. Plasmid pG-Tf2 expresses GroESL and Trigger factor; pTf16 expresses Trigger Factor; pG-KJE8 expresses GroESL and DNA KJE; pGro7 expresses GroESL; pKJE7 expresses DNA KJE. The chaperone plasmids contain the pACYC origin of replication. This origin is in in the p15A compatibility group, while the origins of replication on the plasmids carrying the eCDM8 expression cassette and the Fitness Module are in the pMB1 compatibility group.

### Programmed Evolution Experiments

For use in Programmed Evolution experiments with disks on agar plates, all 24 genotype clones were grown overnight in either LB + 50 µg/mL ampicillin + 35 µg/mL chloramphenicol for clones containing chaperone plasmids or LB + 50 µg/mL ampicillin for clones without chaperone plasmids. Each overnight culture was diluted with LB + 50 µg/mL ampicillin to an absorbance value at 590 nm of 0.025 and equal volumes of the 24 clones were mixed to produce the starting population. A volume of 35 µL of the mixed population was pipetted onto LB agar plates containing 0.05% arabinose and 20 µg/mL tetracycline and distributed with sterile plating beads (Zymo Research). Sterile filter disks (BD Diagnostic Systems no. 231039) were prepared by adding 35 µL of sterile water, 40 mM filter-sterilized theophylline (Sigma-Aldrich), or 40 mM filter-sterilized caffeine (Sigma-Aldrich) and allowing them to stand for 1 minute. Using sterile needles piercing the disks, we placed the disks in the centers of the plates, which were incubated overnight at 37°C and allowed to incubate at room temperature for 4 days.

### Genotype Determination

Determination of chaperones was carried out by colony multiplex PCR. Reactions included 2 µL of an overnight culture of bacteria, 7.5 µL H_2_O, 10 µL 2X GoTaq Green (Promega), and 0.5 µL of primer mix. The primer mix contained the following primers, each at a concentration of 20 µM: Gro_rev 5’ CATCTGCCAGTACGTTTACGC 3’; Gro_for 5’ GAAGAATACGGCAACATGATCGAC 3’; Cm_rev 5’ GATGGTGTTTTTGAGGTGCTCC 3’; Tig_for 5’ TCCGTAGAAGGTCTGCGC 3’; Grp_for 5’ GCGATGGTTACTGTAGCGAAAG 3’. The chaperone PCR thermal profile was 10 minutes at 94°C; 30 cycles of 15 seconds at 94°C, 15 seconds at 51°C, 2 minutes at 74°C; 5 minutes at 74°C. PCR products sizes for chaperone plasmids were 1394 bp for pGro7, 598 bp for pG-KJE8, 1220 bp for pKJE7, 1869 bp for pTF16, and 1706 bp for pG-Tf2. PCR products were analyzed on either 2% agarose gels in TAE or 3.5% polyacrylamide gels in TBE with reference to a marker (All Purpose Hi-Lo DNA Marker, Bionexus, Inc.). The origins of replication were genotyped by multiplex PCR with four primers. Two primers bracketed both of the origins. The other two primers bound to either the high copy HS-A origin or the low copy IT origin. The origin-bracketing primers were 1A2_310_rev 5’ caaaaggccagcaaaagg 3’ and 1A2_310_for 5’ GCTTTTTTGCACAACATGGG 3’. The high copy number primer was 1A2_for 5’ GCGCAGATACCAAATACTGTT 3’ and the low copy primer was IT_reverse 5’ ggctacactagaaggggt 3’. Reactions included 2 µL of an overnight culture of bacteria, 7.6 µL H_2_O, 10 µL 2X GoTaq Green (Promega), and 0.4 µL of primer mix. The primer mix contained the four primers, each at a concentration of 20 µM. Touchdown PCR was needed, with a thermal profile of 10 minutes at 94°C; 20 cycles of 15 seconds at 94°C, 15 seconds at 64.5°C decreasing 1 degree each cycle, 1.25 minutes at 74°C; 20 cycles of 15 seconds at 94°C, 15 seconds at 44.5°C, 1.25 minutes at 74°C; 5 minutes at 74°C. The high copy PCR product of 500 bp and the low copy PCR product of 750 bp were analyzed on 1% agarose gels in TAE by reference to a marker (All Purpose Hi-Lo DNA Marker, Bionexus, Inc.).

### LCMS Measurement of Theophylline Production

We measured theophylline production in the three most fit and two of the least fit bacterial strains using LCMS analysis. All solvents used were of analytical grade. Solutions were prepared with distilled, ultra-pure water (Barnstead Easy Pure II). Standards of caffeine and theophylline were obtained from Sigma Aldrich. Ethyl acetate, acetonitrile and methanol (HPLC grade) were purchased from Fisher Scientific. Bacteria were grown on plates containing 4 mM caffeine, 0.05% arabinose, but no tetracycline overnight at 37°C and allowed to incubate at room temperature for 4 days. Cells were harvested and transferred to 2.0 mL centrifuge tubes containing ultra-pure water (0.5 mL). The tubes were capped and vortexed for 0.5 minutes. Ethyl acetate (0.5 mL) was added to each tube and the tubes were vortexed for an additional 5 min. After centrifugation at 14,000 x g for 5 minutes, 0.5 mL aliquots from each sample were transferred to clean 2.0 mL centrifuge tubes. Samples were dried overnight, resuspended in 1 mL of 9:1 water: acetonitrile. The DNA concentration of a 3 µL aliquot of the resuspension was measured on a NanoDrop 1000 using absorbance at 260 nm. Samples filtered via syringe through a 0.22 µm syringe driven filter unit (Millipore Millex-GV) in preparation for LCMS analysis. Samples were analyzed using a Waters Acquity ultra performance liquid chromatography (UPLC) system equipped with a Chromegabond WR C18 15 cm x 2.1 mm, 3 µm particles, and 120 Å pore analytical column (ES Industries). The UPLC system was interfaced to a Waters Quattro Premier XE triple quadrupole mass spectrometer. A binary gradient solvent was used as the mobile phase: solvent A = water with 0.1% formic acid, solvent B = acetonitrile with 0.1% formic acid. The total run time was 20 min at a flow rate of 200 µL/min. The injection volume was 5 µL. The gradient profile was as follows: time 0 min = 5% B, time 2.0 min = 5% B, time 10.0 min = 30% B, time 10.1 min = 90% B, time 15 min = 90%B, time 15.1 min = 5% B, time 20.0 min = 5% B. Mass spectra were obtained using positive ion electrospray ionization (3.0 kV) in multiple reaction monitoring (MRM) mode. The collision gas was argon and the collision cell pressure was 3.2 E-3 mbar. Theophylline was measured by monitoring transitions 181>124 m/z with a cone voltage of 35 V, collision energy of 19 eV and a dwell time of 0.1 sec. Caffeine was measured by monitoring transitions 195>138 m/z with a cone voltage of 40V, collision energy of 20 eV and a dwell time of 0.1 seconds. For theophylline quantification, a standard calibration curve was generated using theophylline concentrations of 600 ppb, 60 ppb and 12 ppb. Standards were measured in duplicate.

### Statistical Analysis

To compare the three biological replicates of the Programmed Evolution experiments on plates, we used the statistical package R [28] to conduct a two-sided Fisher’s Exact Test on the counts for the three plates under the null hypothesis that the distribution of clones on each of the three plates represented the same distribution of evolved strains. Under this null hypothesis, it is highly likely (*p* = 0.66) to obtain distributions as different from each other, or more so, than what was observed. Therefore we cannot reject the null hypothesis, and we have confidence in treating all 268 clones obtained from all three plates as observations in a single experiment. To compare the observed genotype distribution of the 268 clones with the discrete uniform distribution across all 24 genotypes, we used a chi-squared goodness of fit test. The chi-squared statistic was 2076 (23 degrees of freedom), corresponding to a p-value of less than 10^–300^. We used exact probabilities from the binomial distribution to find the p-values for the observed number of clones with the low-copy origin of replication under the null hypothesis that the high and low copy numbers are equally abundant. The p-value was calculated in the same way for the observed number of clones with the strong promoter and RBS (under the null hypothesis that the two possibilities were equally abundant) for the observed number of clones with the combination of strong promoter and RBS with the low-copy origin of replication (under the null hypothesis that all four combinations were equally abundant) and for the observed number of clones with a chaperone (under the null hypothesis that 5/6 of the population has a chaperone).

## Results

### Combinatorics Module

Standardization of DNA assembly is an important component of synthetic biology [29]. Standardized DNA assembly allows practitioners to design and build interchangeable parts that can be shared between labs. The BioBrick method, developed by Tom Knight, uses the type II restriction enzymes XbaI and SpeI, which produce compatible sticky ends [27]. Adjacent DNA parts are ligated via XbaI and SpeI compatible ends, producing an uncuttable six-base scar after ligation. Golden Gate Assembly (GGA) is an alternative to BioBrick assembly [24,30]. It relies on type IIs enzymes, such as BsaI, that bind to an asymmetrical DNA sequence and produce a sequence-independent, staggered cut at a specific distance from the binding site. Two DNA parts to be connected by GGA are engineered to have type IIs restriction sites that produce complementary sticky ends [31]. The two DNA parts are mixed with type IIs restriction enzyme and DNA ligase in a single reaction. Ligation of the two DNA subparts results in an end product devoid of the type IIs restriction sites with no scar separating the two joined parts.

**Fig 3:**
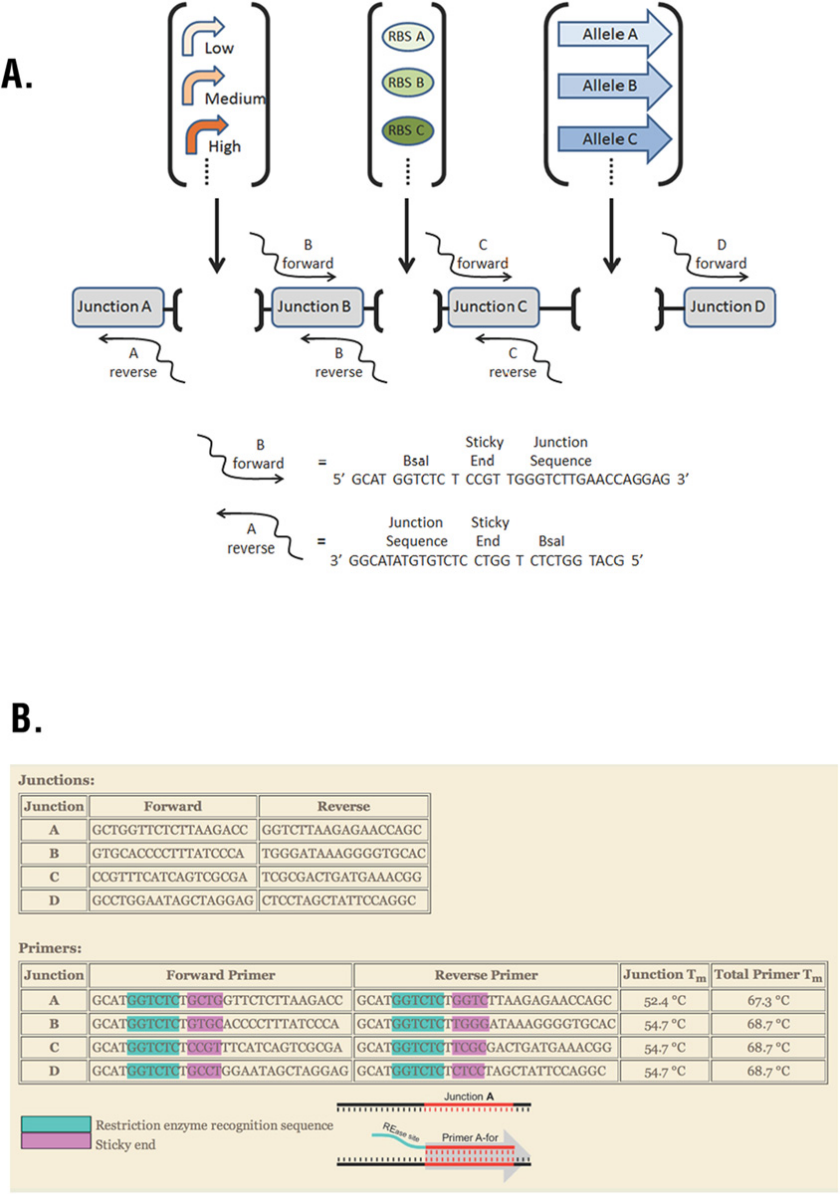
Combinatorics Module. (A) Junction-Golden Gate Assembly (J-GGA) introduces genetic variation into a single gene expression cassette as shown, or multiple gene expression cassettes arranged in tandem. PCR amplifies the vector and adds BsaI restriction sites and sticky ends complementary to the elements to be inserted. J-GGA inserts element(s) using standardized PCR primers regardless of the insert sequences. (B) The online Golden Gate Assembly Junction Evaluative Tool (GGAJET) enables users to design junctions with compatible sticky ends and specific primers with similar melting temperatures. GGAJET is available at gcat.davidson.edu/SynBio13/GGAJET/.

The Combinatorics Module for Programmed Evolution is an adaptation of GGA that enables high throughput introduction of genetic variation from libraries of DNA parts into one or more expression cassettes on a single plasmid. We called our modification Junction Golden Gate Assembly (J-GGA) because the cloning takes place in a scaffold that has junction sequences flanking each element of a gene expression cassette (Fig. 3A). Four junctions form the scaffold for a single gene expression cassette. J-GGA insertion of a library of DNA parts begins with inverse PCR of the plasmid using primers that flank the location of the element to be cloned. PCR results in amplification of the entire plasmid except the element or elements being to be cloned (Fig. 3A). The primers carry BsaI sites that produce unique sticky ends. For example, introduction of promoter variation requires PCR of the plasmid with the B forward and A reverse primers. The promoter library of inserts can be generated by DNA synthesis of top and bottom strand oligonucleotides that can be annealed to produce the sticky ends for junctions A and B. Alternatively, the promoter library can be produced by PCR of cloned promoters using primers that carry BsaI sites that produce the sticky ends for junctions A and B. The plasmid and inserts are ligated by GGA and transformed into competent cells. Because each junction is a unique sequence, multiple families of genetic elements can be cloned simultaneously as described [24].

If large amounts of genetic variation are introduced simultaneously into multiple genetic elements in one or more gene expression cassettes controlling orthogonal metabolism, the number of combinations may exceed the number of bacteria in the population. Exploration of the solution space can be carried out in stages by successive cycles of Programmed Evolution. For example, variation in the RBS could be introduced into a population whose plasmids had previously undergone Programmed Evolution involving promoter variation. Plasmid preparation by inverse PCR would use C forward and B reverse primers. The RBS library with appropriate sticky ends could be generated by DNA synthesis or PCR. A third cycle of Programmed Evolution could involve variation in the alleles, protein degradation tags, plasmid copy number or additional promoter and RBS variants. J-GGA could be used to introduce variation into each of the elements individually or multiple elements simultaneously. For example, all three of the promoters for a series of three different gene expression cassettes could be varied at once by choosing primers and sticky ends for the appropriate junctions. Variation could also be introduced into combinations of elements, such as the promoter for the first gene, the RBS for the second gene, and the allele for the third gene.

A potential problem with simultaneous variation of multiple elements in J-GGA is dimerization of primers used to produce the sections to be assembled. Another concern is non-specific binding of fragments during GGA ligation because of similarities among the set of sticky ends. The latter constraint is particularly limiting in light of the preference for three or more Gs or Cs to ensure annealing of sticky ends during assembly. Only eight 4-base oligonucleotides with 3 or more Gs or Cs are mutually compatible, so the number of elements that can be simultaneously varied and reassembled under the above restrictions is limited to four. We have written software called Golden Gate Assembly Junction Evaluative Tool (GGAJET; [25]) that enables users to design junctions with compatible sticky ends and specific primers. GGAJET also screens for primer self-dimerization and chooses primers with similar melting temperatures. GGAJET can be used in a batch mode, generating up to four junctions at a time, or in iterative mode, testing and adding one junction at a time to an existing set, up to a maximum of eight junctions (Fig. 3B).

For use in a trial run of the Combinatorics Module, we used GGAJET to design a scaffold for use in J-GGA and entered it in the Registry of Standard Biological Parts as J119314. We prepared the P5 promoter (J119031) by annealing synthetic oligonucleotides and successfully ligated it with J-GGA into the scaffold prepared by amplification with the B forward and A reverse PCR primers. We also used J-GGA to insert a bicistronic RBS (J119024; [23]) between Junction B and Junction C. The primers carried BsaI sites and the appropriate sticky ends to insert the RFP gene between Junction C and Junction D. The final product was verified by RFP expression and DNA sequencing.

### Plasmid Copy Number as a Genetic Variable

In addition to promoters, RBSs, and alleles, plasmid copy number (PCN) can be used as a genetic variable in Programmed Evolution. The number of plasmids maintained per cell affects the number of mRNA transcripts produced for the synthetic circuit. Furthermore, the cell expends energy to maintain plasmid copy number, which is one of the analog inputs bacteria factor into Programmed Evolution. Resources used for transcription and translation of metabolic enzymes have unknown effects on the ability of cells to perform the desired orthogonal metabolism and compete with other cells in the population. Plasmids with high, medium, and low copy number are available in the Registry of Standard Biological Parts [32]. To expand the options for plasmid copy number, we developed a method to alter the output of well-known origins of replication. As a target for copy number engineering, we chose the high copy number origin of replication in the family of vectors derived from pSB1A2. This origin of replication is derived from the pMB1 origin of replication.

**Fig 4:**
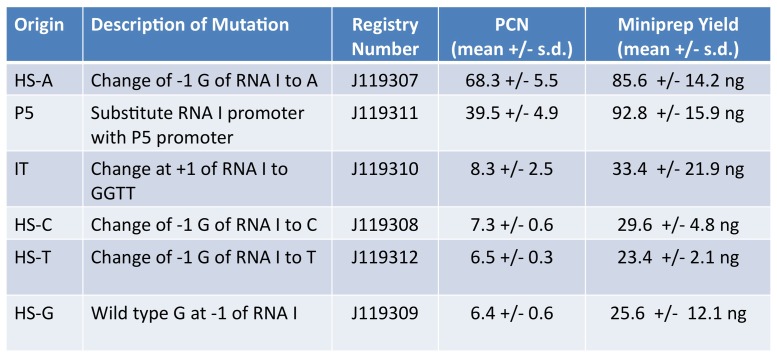
Origins of Replication Determine Plasmid Copy Number. The origins of replication used in the study are listed with their descriptions and part numbers in the Registry of Standard Biological Parts. The means and standard deviations of PCN values were determined by qPCR and yields of minipreps.

The pMB1 origin of replication encodes two transcripts, RNA I and RNA II, which are encoded by overlapping genes in opposite orientations [26]. RNA II is processed into a primer used for the initiation of replication. RNA I is antisense to a large portion of RNA II, and when the two hybridize, RNA II cannot be used as a primer. As a result, increased transcription of RNA I reduces the copy number of pMB1 plasmids. We designed and measured the effect of pMB1 mutations on PCNs. Based on published work [26], we changed the hotspot base at the-1 position of the RNA I gene from G to each of the other three bases, and changed the first four bases of RNA I from ACAG to GGTT. We also replaced the endogenous RNA I promoter with a synthetic inducible promoter. We used qPCR to measure PCN with a pair of primers that bind to the ampicillin resistance gene on the plasmid and a pair of primers that bind to the chromosomal copy of the DNA polymerase I gene. PCNs were determined by comparing the amplification of the plasmid and chromosomal DNA [26]. We determined the average PCNs of five mutant origins HS-A, HS-T, HS-C, HS-G, P5, and IT (Fig. 4). To validate the qPCR results, we performed multiple plasmid mini-preps and quantified DNA yield. Mutation of the-1 hotspot from G to A (HS-A) produced the highest PCN. When the-1 hotspot base is C, T, or G, the PCN is at or near the lowest level among the mutants. The IT mutation, which changed the first four bases of the RNA I gene, also reduced the PCN. The sequence of the pMB1 origin recorded for pSB1A2 in the Registry of Standard Biological Parts (http://parts.igem.org/Part:pSB1A2) includes a G at the hotspot. However, the pSB1A2 origin that we used contained the HS-A mutation. We sequenced six other pSB1A2 clones from the Registry dating back to 2009, and they all contained the-1 hotspot A mutation. We deduced that the hotspot A mutation increased PCN and has been unknowingly maintained in our labs by selection. The mutation that resulted in the second highest PCN resulted from replacement of the wild type RNA I promoter with a strong P5 promoter. We used J-GGA to produce a library of PCN variants for Programmed Evolution and added these parts to the Registry of Standard Biological Parts (Fig. 4).

### Biosensor and Fitness Modules

Fitness and Biosensor Modules for Programmed Evolution must be capable of binding the desired metabolic product. Riboswitches are attractive candidates for this function because they are RNA-based controllers of gene expression that use aptamers to bind a wide range of ligands [33]. Riboswitches are most often found in the 5’ untranslated regions of naturally occurring mRNAs. Riboswitches change conformation upon ligand binding in ways that can affect the efficiency of transcriptional termination, the accessibility of the RBS for initiation of translation, or cleavage of the mRNA by ribozyme activity [33]. Using Systematic Evolution of Ligands by Exponential enrichment (SELEX), RNA aptamers for use in riboswitches can be generated *de novo* from randomized RNA oligonucleotides through selection for ligand binding followed by PCR amplification [34].

**Fig 5:**
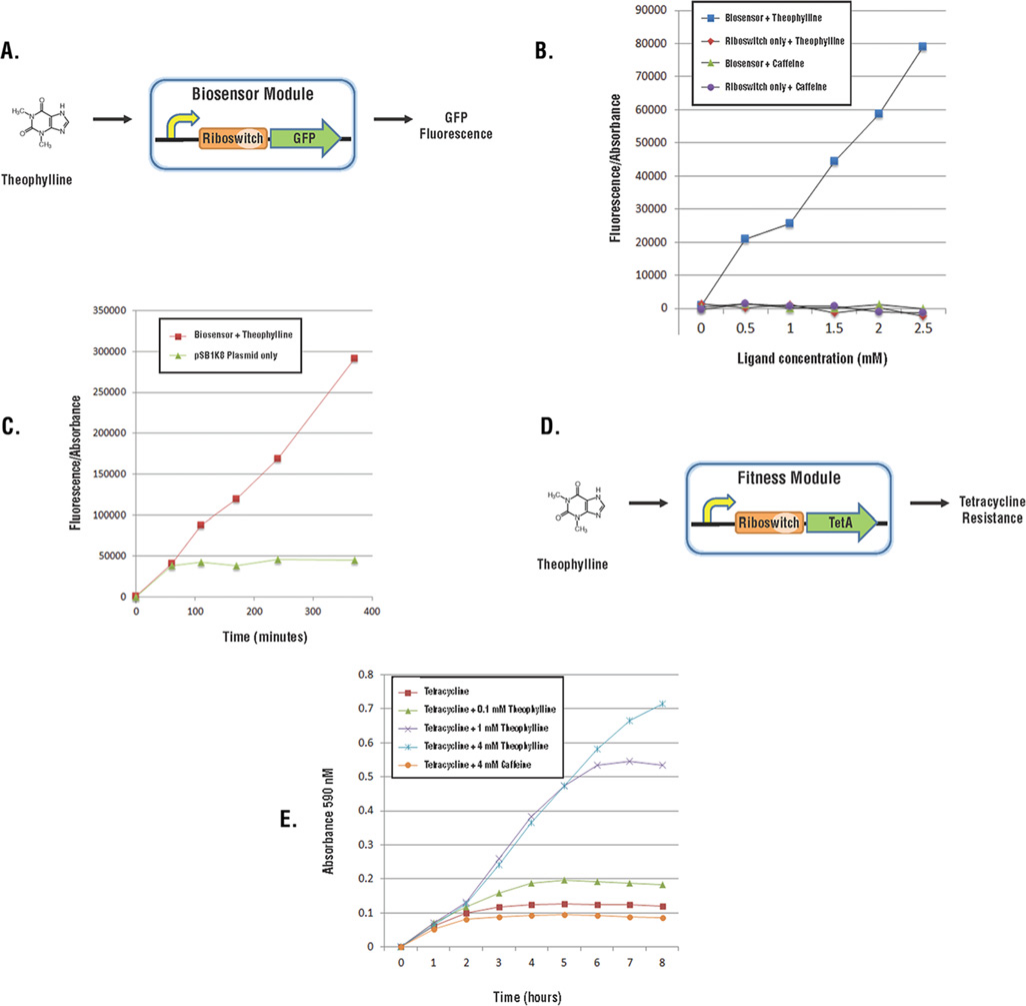
Biosensor and Fitness Modules. (A) The Biosensor Module contains a promoter, a riboswitch that binds to theophylline, and a GFP gene. (B) Cells with the indicated genotypes were incubated with caffeine or theophylline. Fluorescence of cells grown in theophylline or caffeine was divided by absorbance at 590 nm (relative fluorescence) to correct for variation in cell density. (C) Relative fluorescence as a function of time in cells with and without the biosensor grown in 2.5 mM theophylline. (D) The Fitness Module contains a promoter, a riboswitch that binds theophylline, and the tetracycline resistance gene (*tetA*). (E) Cell growth in media containing tetracycline and either theophylline or caffeine as indicated.

For proof-of-concept, we built a theophylline Biosensor Module using the well-characterized theophylline riboswitch D [35] to regulate the production of GFP or RFP in a time- and dose-dependent manner (Fig. 5A). The riboswitch is responsive to theophylline but unresponsive to caffeine (Fig. 4B and 4C). In detailed experiments using 0 to 0.5 µM theophylline, we found the biosensor responded equally well between 0.1 and 0.5 µM but there was a linear decline in biosensor response between 0 and 0.1 µM theophylline. Because aptamers can be developed for nearly any metabolite [33], the Biosensor Module could be engineered for almost any desired product.

Metabolic engineering of bacteria is at odds with the natural evolution of cell populations [3]. Cells that reduce their output of the desired product are favored by natural selection because the reduced energy drag they experience results in higher fitness [36]. Over time, a subpopulation of cells will evolve to reduce product formation because they experience no reward for product formation and no punishment for its reduced production. Soon, the population is dominated by non-productive cells and the desired metabolite is no longer produced. Rather than fight natural selection and evolution, we decided to harness the evolutionary mechanism of natural selection to maintain maximum production of the desired product in a population. To convert natural selection from an enemy to an ally, we developed the Fitness Module, which rewards cells for producing the desired product by increasing their fitness. The Fitness Module also ensures that the bacterial population maintains the genotype or genotypes optimal for the desired metabolic output.

The Fitness Module contains a riboswitch upstream of a fitness gene. A Fitness Module can function in a variety of ways depending on the fitness gene employed. For the production of theophylline, we tested the riboswitch upstream of the *E*. *coli adhE* gene, which codes for alcohol dehydrogenase [37]. Using an *adhE* ^-^ strain with the nonessential *adhE* gene deleted, we intended to grow the transformed cells in media containing ethanol as the only energy source. In this way, fitness should be determined by the ability of cells to convert caffeine to theophylline. However, *adhE* ^-^ cells were able to grow with ethanol despite published reports to the contrary [37]. We have been more successful with a Fitness Module that has the riboswitch upstream of the *tetA* gene, which encodes tetracycline resistance (Fig. 5D). The fitness module shows a dose-dependent response to theophylline but is unresponsive to caffeine (Fig. 5E).

### Programmed Evolution of Theophylline Production

We chose the conversion of caffeine to theophylline as our proof-of-concept of Programmed Evolution for several reasons. Theophylline is a common treatment for asthma, so it has a practical application [38]. Producing theophylline from caffeine is a one-step metabolic pathway, so only one gene expression cassette was required [8]. *E*. *coli* does not produce theophylline on its own, which minimized the possibility that cells would produce the desired compound without our synthetic gene circuit. The caffeine demethylase enzyme (*yCMD8*) was engineered through directed evolution to function in yeast cells and does not occur in *E*. *coli* [8]. Finally, a theophylline riboswitch was readily available and functions very well with regard to its specificity and dynamic range [39].

**Fig 6:**
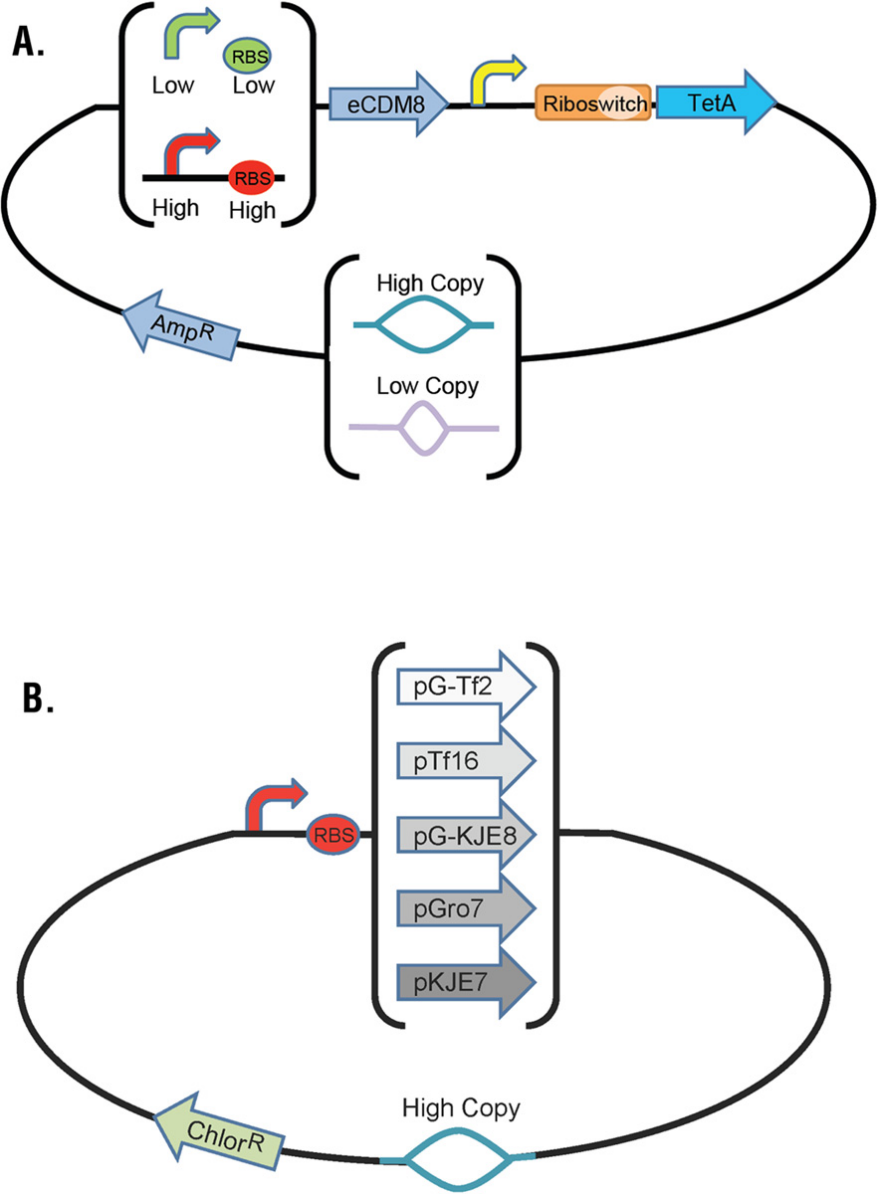
Starting Population for Programmed Evolution. (A) An ampicillin resistance plasmid carries variation in the strength of promoters and RBS elements as well as the low and high copy number origins of replication. (B) A chloramphenicol resistance plasmid carries chaperones DNA KJE, Trigger Factor, and Gro ESL chaperones individually and in two combinations (see Methods for details).

The optimization of theophylline production in *E*. *coli* is likely to depend on many known variables associated with the control of transcription, translation, enzyme structure and function, and metabolic flux, as well as many variables that cannot be known. We decided on three genetic variables for use in our Programmed Evolution proof-of-concept experiments. We manipulated promoter and RBS strengths in the caffeine demethylase expression cassette and the PCN variable (Fig. 6A). We also included five different chaperone expression plasmids widely used with the goal to improve expression of orthologous proteins in *E*. *coli* (Chaperone Plasmid Set, Clonetech Laboratories, Inc.). Chaperone plasmids encoded the DNA KJE, Trigger Factor, and GroESL chaperones individually and in two combinations on a plasmid encoding chloramphenicol resistance and a pACYC origin of replication. (Fig. 6B). We cloned the theophylline Fitness Module and the caffeine demethylase expression cassette on an ampicillin resistance plasmid (Fig. 6A). To facilitate genotyping cells carrying the high strength promoter and RBS, we added an RFP expression cassette downstream of the Fitness Module. The low strength promoter and RBS carried a GFP expression cassette downstream of the Fitness Module. We used two promoter + RBS variants, two PCN variants, and six chaperone choices (five chaperones plus the case with no chaperones) to produce 24 different strains of *E*. *coli* for testing Programmed Evolution.

**Fig 7:**
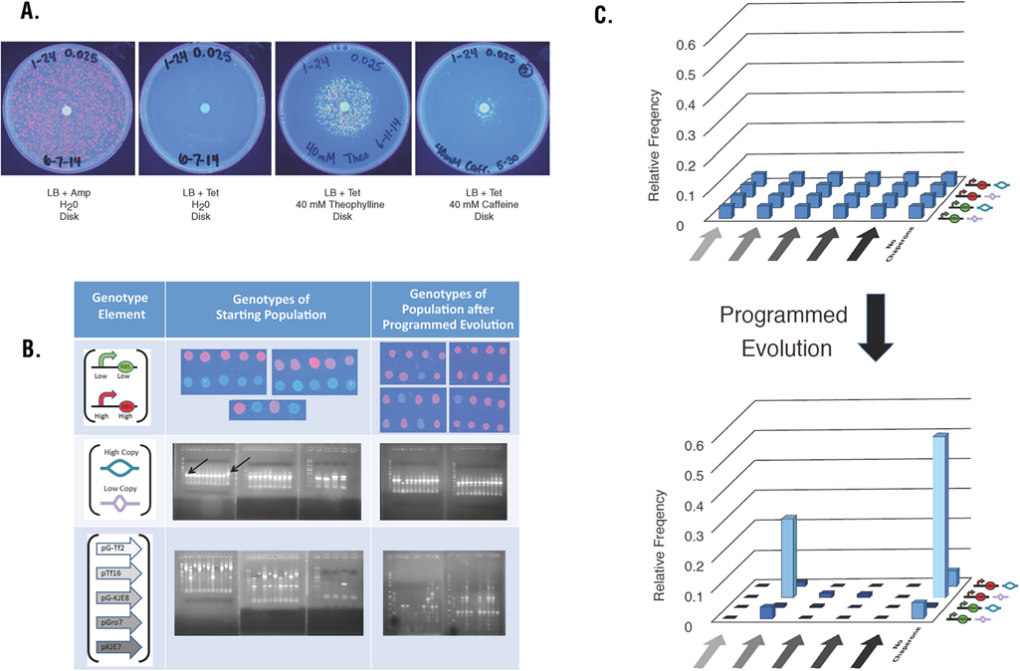
Results of Programmed Evolution. (A) The starting population with equal amounts of all 24 strains was spread on LB agar plates with the indicated antibiotic and a disk treated as indicated. (B) Top row: spots of cells on LB agar with ampicillin for all 24 starting strains (left) and examples of clones after Programmed Evolution (right). Middle row: Agarose gels with PCR products to determine PCN for all 24 strains (left) and examples after Programmed Evolution (right). The 750 bp band for the low copy origin and the 500 bp band for the high copy origin are indicated by arrows. Bottom row: Agarose gels with PCR products to chaperone genotype for all 24 strains (left) and examples after Programmed Evolution (right). (C) The graph shows relative frequency of each of the genotype before (top) and after (bottom) Programmed Evolution. The order of chaperone plasmids along the left to right horizontal axis is pG-Tf2, pTf16, pG-KJE8, pGro7, pKJE7, and no chaperone. The order of genotype combinations along the other horizontal axis from back to front is high strength promoter/RBS + high copy origin; high strength promoter/RBS + low copy origin; low strength promoter/RBS + high copy origin; and low strength promoter/RBS + low copy origin.

We conducted Programmed Evolution on agar plates with caffeine disks because we did not know the optimal concentration of caffeine to use in the experiments. We put 40 mM caffeine on sterile filter disks and allowed diffusion to produce a concentration gradient of caffeine on the plates (Fig. 7A). The plate on the left contained ampicillin and a disk soaked in water, and was seeded with the population of 24 strains mixed in equal proportions. The second plate contained the same mixed population of 24 strains on a plate with tetracycline and a water disk. None of the bacterial clones grew because there was no theophylline to activate the Fitness Module encoding tetracycline resistance. The third plate contained the same population of 24 strains on a tetracycline plate with a disk containing 40 mM theophylline. Activation of the Fitness Module by theophylline explains the visible ring of growth around the disk. The fourth plate contained the 24 strain population seeded onto a tetracycline plate with a disk containing 40 mM caffeine. Because a high concentration of caffeine is toxic to cells, we saw a zone of inhibition adjacent to the disk. The narrow zone of growth indicates that bacteria are able to use caffeine demethylase to convert a narrow concentration range of caffeine to theophylline and turn on the Fitness Module that enables them to grow in the presence of tetracycline.

Multiple negative control plates with water disks and positive control plates with theophylline disks support the conclusion that colonies growing on plates with caffeine disks carry genotypes that provide them with fitness as defined by the theophylline Fitness Module. Bacterial strains that carried only the Fitness Module, only the caffeine demethylase expression cassette, or neither of these were repeatedly unable to grow on the plates with caffeine and tetracycline. To detect changes in the frequency of population genotypes after Programmed Evolution, we determined the frequency of colony genotypes for cells growing in the ring around the caffeine disk. The combined promoter and RBS variable was determined by spotting clones on plates and looking for either RFP expression indicating strong promoter and RBS, or GFP expression indicating weak promoter and RBS. Fig. 7B verifies that half of the 24 clones in the starting population produced RFP and half produced GFP. After Programmed Evolution, the percentage of RFP colonies increased to 82.7%, indicating that the genotype distribution in the viable population had shifted toward the strong promoter and RBS. We used PCR to determine the type of origin of replication carried by colonies containing the optimization solution. Plasmids with a high PCN produced a 750 bp PCR product and those with a low PCN produced a 500 bp PCR product. Fig. 7B shows that 50% of the starting population carried the high copy number plasmid and 50% carried low copy number plasmids. After Programmed Evolution, the distribution of PCN is strongly shifted in favor of low PCN (91.4%). We also used PCR to detect and characterize chaperone plasmids. Each of the chaperone plasmids produced a unique banding pattern, as seen in the gel photographs in Fig. 7B. Four clones in the starting population did not contain chaperone plasmids, but PCR using those strains as templates resulted in a nonspecific product of about 750 bp that distinguished the no chaperone genotype from cells carrying a chaperone plasmid. The starting population contained all the expected chaperones. After Programmed Evolution, most of the colonies growing in response to the caffeine disk did not contain a chaperone plasmid (64.9%), but when a chaperone plasmid was present, it was usually pTf16 (91.5% of those with plasmids). The lack of chaperone plasmids in two-thirds of the colonies was confirmed by the inability of bacteria to grow in the presence of chloramphenicol.

**Fig 8:**
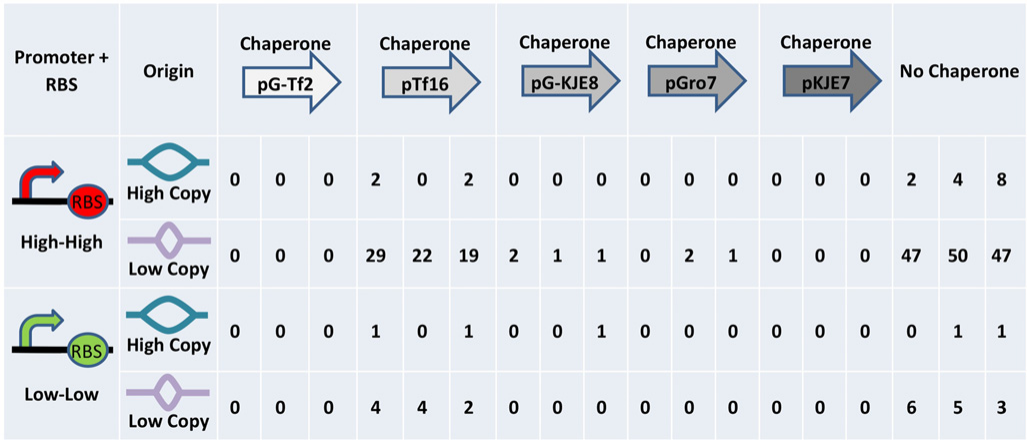
Results of Programmed Evolution. The number and genotype of colonies analyzed after Programmed Evolution from three replicate plate experiments.

We began Programmed Evolution by seeding three tetracycline plates as biological replicates with mixed populations containing equal amounts of all 24 strains and placing a caffeine disk in the center of each plate. At the end of the experiment, we picked 96 colonies off of each plate within a growth ring of cells surrounding the disks (far right plate in Fig. 7A). All 288 of the picked colonies were located within a diameter of four times the disk diameter. Each of the picked colonies was incubated overnight in LB broth with ampicillin, and 268 of the 288 clones grew. Promoter and RBS genotype was determined by spotting on plates and observing fluorescence. PCN and chaperone genotypes were determined by PCR and gel electrophoresis (Fig. 7B). Fig. 8 shows the distribution of genotypes among the 268 viable clones. The distribution of evolved genotypes is very similar on each of the three biological replicate plates (*p* = 0.66), allowing us to treat the 268 clones as members of a single population. Fig. 7C shows a graphical representation of the change in distribution of genotypes in the population before and after Programmed Evolution. Increased fitness of individual genotypes causes towers to rise from the background relative frequencies of the majority of the members of the population. The expected occurrence of a given genotype in the absence of evolution is 11 out of the 268 clones. The most fit strain contained a strong promoter and RBS, a low PCN, and no chaperone, and occurred 144 times among the 268 clones. The second most fit strain also contained a strong promoter and RBS and the low PCN, along with chaperone Tf16, and occurred 70 times out of 268 clones. Only two other genotypes occurred more often than the expected occurrence in the absence of evolution (11 out of 268). The third and fourth most common genotypes were the strong promoter and RBS with high PCN and no chaperone (14 out of 268) and the weak promoter and RBS with low PCN and no chaperone (14 out of 268). These two combination occurred only slightly more often than the expected occurrence in the absence of evolution of 11 out of 268 clones. The genotype of weak promoter and RBS with low PCN and chaperone Tf16 occurred 10 times out of 268 clones. Six additional genotypes occurred between 1 and 4 times, and 13 genotypes (more than half the 24 original genotypes) are not detected at all among the 268 viable clones. The distribution of genotypes in the 268 clones is significantly different from the uniform distribution across all 24 genotypes (p < 10^–300^). Our results demonstrate that Programmed Evolution significantly changed the distribution of genotypes in the bacterial population.

**Fig 9:**
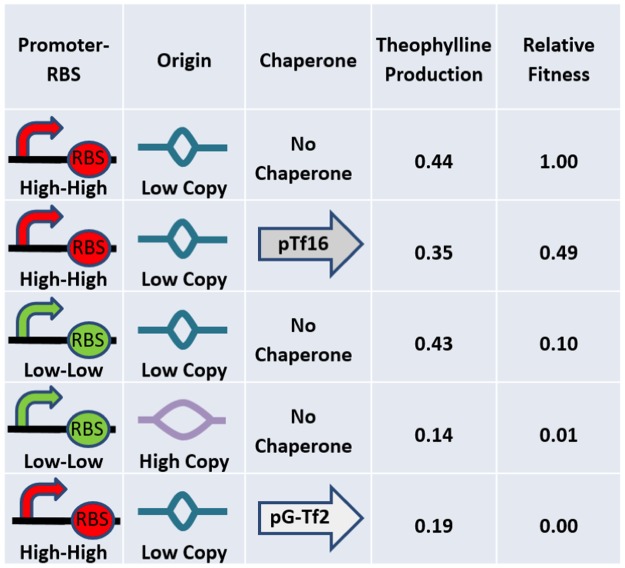
Relative Fitness of Genotypes as a Function of Theophylline Production. Theophylline production as measured by LC-MS analysis is listed for the three genotypes with the highest fitness and two genotypes with very low fitness.

To demonstrate that Programmed Evolution was successful in selecting the optimal genotype for the sustained conversion of caffeine to theophylline by caffeine demethylase in *E*. *coli*, we measured theophylline levels in several of the strains using LCMS. Fig. 9 lists theophylline production by the top three highly fit genotypes and two of the least fit genotypes, all of which contained the caffeine demethylase gene. Each strain was grown on plates containing 4 mM caffeine but no tetracycline. Bacteria harvested from the plates were extracted for LCMS analysis of theophylline concentration and NanoDrop measurement of DNA concentration. Theophylline concentrations in the bacterial extracts ranged from 28 to 80 ng/µL. DNA concentrations ranged from 94 to 220 ng/µL. Genotype-specific theophylline production was calculated as the ratio of the theophylline concentration to the DNA concentration. Relative fitness in Fig. 9 was calculated by dividing the number of colonies for each genotype by the number of colonies for the most fit genotype. The results show a two- to three-fold higher level of theophylline in the three most fit genotypes compared to the least fit genotypes.

Close examination of the far right plate in Fig. 7A reveals colonies growing outside the growth ring of cells surrounding the caffeine disk. Because this effect was reproducible, we investigated the genotype of these outside colonies. Of 31 outside colonies on three biological replicate plates, 71% were one of the two genotypes found most often inside the growth ring. Fifteen of the 31 outside colonies analyzed contained a strong promoter and RBS, the low PCN, and no chaperone; 7 contained a strong promoter and RBS and the low PCN, along with chaperone Tf16. Only four other genotypes occurred outside the growth ring, with occurrences of 4, 2, 2, and 1, respectively. These results are in accord with the results from the growth ring of cells surrounding the caffeine disk.

As an extension of the results shown in Fig. 7, we produced agar plates containing tetracycline and concentrations of caffeine varying from 0 to 20 mM and seeded them with equal numbers of the two most fit genotypes. In these experiments, plates with 1 to 4 mM caffeine produced about 1500 colonies each, but plates with 5 mM or higher concentrations of caffeine produced fewer than 100 colonies. Therefore, we conclude that the ring of growth in Fig. 7A occurred where the caffeine had diffused to a local concentration of 1 to 4 mM caffeine.

## Discussion

### Variables Affecting Optimization

Bacterial production of economically valuable compounds is a foundational practice in pharmaceutical, energy and other industries. However, large-scale growth of bacteria and sustained production of the product suffers when the populations evolve away from producing the desired output. To address this critical problem, we designed and implemented Programmed Evolution. Programmed Evolution takes advantage of the natural computing power of bacteria, and harnesses evolution as a tool for optimization.

Our first application of Programmed Evolution involved optimizing theophylline production by caffeine demethylase. The specific metabolic product of our successful application is less important than the proof-of concept of our approach. Rather than guessing *a priori* which combination of genetic variables we thought would be best for *E*. *coli* to thrive while producing the desired product, we programmed it with 24 possible genetic solutions and let the its inherent analog computational power integrate the dynamic response capacity with the consequences of the encoded synthetic genetic circuit. We manipulated three variables and produced 24 different *E*. *coli* strains as the starting population of possible optimization solutions. The distribution of genotypes after Programmed Evolution revealed the relative importance of each of the variables for fitness as defined by the Fitness Module. Unexpectedly, PCN had the largest effect on fitness. Of the 268 viable clones isolated after Programmed Evolution, 245 (91%; *p* = 2.4x10^–48^) had the low copy number origin. The variable that had the second largest effect on fitness was the strength of the combined promoter and RBS control elements. Of the 268 clones, 239 (89%; *p* = 1.5x10^–42^) carried the strong promoter and RBS (Fig. 8). Of the viable clones, 221 (82%; *p* = 8.9x10^–87^) carried both the low copy number origin and the strong promoter and RBS.

The increased fitness associated with a small subset of the 24 possible genetic control element combinations may be due to a balance between using cellular resources for plasmid replication and using them for caffeine demethylase expression and theophylline production. The level of caffeine demethylase can be abstracted as a mathematical function that is equal to the copy number times the level of gene expression. Populations will evolve to favor cells that balance the cost of maintaining the synthetic circuit with the benefits of surviving the selection imposed by the Fitness Module. If cells only need *x* caffeine demethylase enzymes per cell to survive selection, our results suggest that it is preferable to maintain a small number of plasmids that direct high levels of caffeine demethylase production rather than producing more plasmids that direct a low level of caffeine demethylase. We produced a population of 24 different strains that had different ways to produce *x* caffeine demethylase enzymes per cell, but 82% of the time, the cells calculated the same optimization solution.

As metabolic engineers, it was comforting for us to think we could “help” the cells by providing them with a range of chaperones to facilitate the folding of an exogenous protein. But chaperones were less important than the other two variables since only 35% (94 of 268) of the viable clones had a chaperone plasmid, significantly fewer than the 83% of initial clones with a chaperone (*p* = 4.6x10^–68^). Chaperones had only a moderate effect on fitness, but the choice of chaperones clearly mattered. Of the 94 clones that had chaperones, 91% (86 of 94) computed that pTf16 (trigger factor) was the optimal choice. We do not know why this chaperone worked better than the others and perhaps this falls into the category of unknowable variables.

Our measurement of two- to three-fold higher production of theophylline by the three most fit genotypes compared to two of the least fit genotypes is an important validation of our claim that Programmed Evolution optimized production of theophylline in a bacterial population. We view relative fitness as a measure of optimal theophylline production. Careful examination of the theophylline production results presented in Fig. 9 reveals that relative fitness is not perfectly correlated with maximum theophylline production. We think this is because relative fitness is a function of theophylline concentration over time in the bacteria. The strain containing a weak promoter and RBS, the low PCN, and no chaperone had a relative fitness of 0.10 but produced about the same amount of theophylline as the most fit strain containing a strong promoter and RBS, the low PCN, and no chaperone. The only difference in genotype between these two strains is the strength of the promoter and RBS. A testable hypothesis to explain this observation would be that the strain with the strong promoter and RBS produced high levels of caffeine demethylase that made high levels of theophylline available to the fitness module earlier in cell growth cycle than the strain with the weak promoter and RBS. The results in Fig. 9 also show a lower level of theophylline production in the presence of the trigger factor chaperone (pTf16) than in its absence. This supports the conclusion that relative fitness is a function of optimal of theophylline production, not necessarily maximum. The expression of the trigger factor could involve the use of resources resulting in lower cell viability. Other variables could also affect cell viability, such as the expression of RFP versus GFP in our strains.

Without Programmed Evolution, we could not have predicted which combination of the three genetic variables would have been optimal in *E*. *coli*. To date, no one has been able to accurately predict the fitness impact of a synthetic genetic circuit in bacteria producing dynamic responses to changing environmental and metabolic factors. Programmed Evolution empowers a population of bacteria to compute fitness as a function of all of these variables to produce the optimal outcome of sustained production of the desired metabolite.

### Growth Conditions for Programmed Evolution

We implemented Programmed Evolution of caffeine metabolism on solid media but were unable to do so in liquid media. Our Fitness and Biosensor experiments with clones expressing caffeine demethylase in broth containing caffeine have not resulted in tetracycline resistance or measurable GFP expression, respectively. We experimentally determined that the optimal concentration of caffeine for the top two clones was 4 mM. Programmed Evolution will be more generalizable to other applications if it can be made to function in liquid media. Further investigation of Programmed Evolution using caffeine metabolism and other applications may reveal peculiarities of the caffeine system that explain why our success was limited to disk experiments on agar plates. One possibility is that the caffeine demethylase enzyme may not be functioning well in *E*. *coli*. It was selected to function in yeast cells at 30°C, and the ways in which it is folded, post-translationally modified, and sequestered to a subcellular location may be very different than what occurs in bacteria. Another peculiarity of the caffeine system may be the readiness with which theophylline diffuses out of cells after it is produced by caffeine demethylase. Diffusion of theophylline could prevent a sufficient accumulation of theophylline inside the cells for turning on the tetracycline Fitness Module. A study of yeast cells engineered to secrete and sense a particular signaling molecule revealed how the ratio of self to neighbor communication can affect complex population behaviors [40]. Bacterial cells in our Programmed Evolution experiments secrete theophylline but also sense theophylline. Perhaps our inability to convert Programmed Evolution results from agar plates to broth is related to the observation that yeast cell populations can act as an ensemble to be either quiescent or activated. Finally, it could be that the microenvironment around a colony forming on a plate is required to balance caffeine and theophylline diffusion, caffeine metabolism, and tetracycline resistance in a way that enables cell growth. Colony formation may begin when a given cell is able to get past the tetracycline blockage of translation and make enough of the caffeine demethylase to get to a tipping point. Based on data in this report, we know the biosensor can respond equally to 0.1–0.5 µM theophylline so we suspect the growing colonies were producing enough theophylline to cross the 0.1 µM-threshold of detection and growth. A positive feedback loop could convert caffeine to theophylline and produce enough TetA protein to export tetracycline and increase protein translation, allowing for the production of more caffeine demethylase and more conversion of caffeine to theophylline. The colony could create a microenvironment of theophylline, internally and externally, that is inherited by its descendants. The growing colony might be depleting the local concentration of caffeine, preventing other colonies from being established nearby. For the caffeine system, the formation of a colony in this way may depend on the microenvironment of an agar plate.

### Programmed Evolution beyond the Prototype

We used Programmed Evolution to optimize the conversion of caffeine to theophylline. The bacteria computed the optimal solution from a collection of 24 inputs controlling a single-step metabolic pathway. Programmed Evolution has the capacity to explore a very large, multidimensional space of genotypes governing multistep metabolic pathways. After one cycle of Programmed Evolution optimizes a given variable controlling the desired orthogonal metabolism, subsequent cycles could introduce new elements of genetic variation or revisit elements previously optimized. Variables could include promoter and RBS strengths, chaperone proteins, degradation tags at the ends of proteins, transcriptional terminators between successive gene expression cassettes or different *E*. *coli* host strains. The results from Programmed Evolution involving theophylline production were unexpected. It is highly unlikely that anyone could predict *a priori* the optimal solution to a multistep pathway problem. It is also unlikely that silicon computers could be programmed to solve the optimization problem because too many important variables are unknown or unknowable. We suggest that a better approach is to empower analog bacterial computers to find solutions that are shaped by evolution.

### Programmed Evolution as a Strategy for Metabolic Engineering

Our successful proof-of-concept Programmed Evolution example builds on previous reports of using selection as an approach to metabolic engineering [14,16,17]. Programmed Evolution harnesses natural selection to unleash populations of bacteria that can factor in unknown and unknowable variables that affect orthologous metabolism. We leveraged the inherent analog computational potential of cells in combination with the relentless pressure of natural selection. Programmed Evolution differs from directed evolution, which uses human selection to identify new alleles with improved catalytic capacity, but does not address the problem of sustained optimal output [9,41]. Optimal solutions computed by Programmed Evolution could be adapted for applications in biosynthesis of pharmaceuticals, chemical commodities, and biofuels, or for optimized catabolism in bioremediation [1,3,7,42]. Enzymes for many metabolic pathways either have been or could be expressed in microbes, so the potential applications for Programmed Evolution are as diverse as the collective metabolic map of the biosphere.

Widespread application of Programmed Evolution depends on riboswitches that can be used in Fitness and Biosensor Modules. Riboswitches can bind to a wide range of ligands [33] and can be generated *de novo* from randomized RNA oligonucleotides through selection for ligand binding followed by PCR amplification [34]. A novel strategy for the rational design of synthetic riboswitches uses a combination of *in silico*, *in vitro* and *in vivo* experiments to generate riboswitches that respond to theophylline via antitermination of transcription [43]. Development of new riboswitches is also likely to benefit from the demonstration that the aptamer domain and the expression platform can be effectively decoupled [44]. A set of general design principles for synthetic riboswitches has been described that provides a basis for adapting natural riboswitches for use in synthetic gene regulatory circuits [45]. Although these developments are encouraging, the field of riboswitch development has yet to realize its early promise. Improvements in riboswitch discovery are needed for future applications of Programmed Evolution. The second critical element that needs more attention is new fitness genes for Fitness Modules. We found that *adhE* was not a good gene for use in a fitness module, but we are developing candidate genes under investigation to expand the points at which natural selection can select for optimization of metabolic intermediates. By shifting from one selection pressure to the next, Programmed Evolution could adapt to ever increasing lengths of multi-step pathways.

### Mathematical Modeling of Programmed Evolution

We built and tested combinations of genetic variables in a three-dimensional search space of potential solutions to a metabolic optimization problem. As the number of genotypes increases, we will need a method of determining how many cycles of Programmed Evolution to conduct in order to know when to stop. Mathematically, we view Programmed Evolution as a hybridized human/computer search algorithm for optimizing the dependent variable of metabolic flux as a function of independent variables such as promoter-RBS, PCN, and chaperones. It is easy to imagine a large number of potential values for each of these variables, as well as an arbitrarily large number of additional variables in an expanded search space. Each combination of parts in a particular gene expression cassette, and each combination of such cassettes encoding a multistep metabolic pathway, defines a single point in an *n*-dimensional search space. A set of points is represented by a population of bacterial cells. Humans control the order in which the types of genetic elements will be interchanged in each expression cassette, and the population of cells evaluates the function in a relative sense, by allowing cells to compete with one another using the Fitness Module. The amount of time cells compete, and the strength of the accompanying selection, will determine the frequency of each genotype. Longer times and stronger selection will amplify the fitness differences among the genotypes and reduce the number that survives for subsequent selection.

Mathematical models of Programmed Evolution could guide the human component of the search algorithm. For example, we might assume that degradation tags confer a small amount of variation in fitness, independent of the value of other variables, and that a subset of RBS values are much better than others. We can also model the effect of interaction among variables by using a different range of values for one variable under different settings of the other variables. By choosing a range of situations for each of the variables, we could evaluate different experimental protocols for the human-controlled parts of the search algorithm, and thereby maximize the efficiency with which the cells find the optimal pathway configuration. Comparisons of the ways that silicon and bacterial computers search large spaces for solutions to optimization problems could lead to advances in both computer science and biological engineering.

## Conclusions

We have developed and implemented an approach to metabolic engineering called Programmed Evolution as a dramatically different strategy for the optimization of orthogonal metabolic pathways in bacteria. We report a Combinatorics Module that allows rapid production of genetic variation as a set of possible solutions to difficult optimization problems. We successfully tested a Fitness Module for selection of genotypes that best carry out the desired metabolic pathway to provide sustained output of the desired metabolite, theophylline. We adapted a Biosensor Module to measure metabolic output. We successfully implemented Programmed Evolution to identify 2 optimal genotypes for a genetic circuit controlling theophylline production from a starting population of 24 genotypes. Programmed Evolution could be used to optimize any desired output from genetic circuits encoding metabolic pathways in cells. Programmed Evolution could enable researchers to program cells and use evolution to determine the best combination of genetic control elements for the catabolic destruction of a toxin or the anabolic synthesis of a desired product. Metabolic engineers could use Programmed Evolution for applications in energy, pharmaceuticals, food production, biomining, bioremediation, and more. Programmed Evolution will facilitate plug-and-play modularity that could be applied to any natural or chimeric pathway for which genes that encode the relevant enzymes are sequenced [8]. Our approach offers synergy by integrating the advances in genome editing [11] and DNA synthesis with the persistent selection pressure applied to all living systems.
